# Lower Motor Neuron Disease Misclassified As Inflammatory Myopathy in a Young Woman With Myositis-Specific Antibodies

**DOI:** 10.7759/cureus.116583

**Published:** 2026-09-20

**Authors:** Khoa Quy, Ai-Thao-My Doan, Thi-Mai Vu, Thi-Minh-Phuong Nguyen, Dinh Nguyen

**Affiliations:** 1 Health Sciences, VinUniversity, Hanoi, VNM; 2 Allergy and Clinical Immunology, Vinmec Times City International Hospital, Hanoi, VNM; 3 Neuroscience, Vinmec Times City International Hospital, Hanoi, VNM; 4 Immunology, VinUniversity, Hanoi, VNM; 5 Medicine, The Pennsylvania State University, Pennsylvania, USA; 6 Medicine, The University of Sydney, Sydney, AUS

**Keywords:** diagnostic anchoring, genetic analysis, idiopathic inflammatory myopathies, lower motor neuron weakness, muscle biopsy findings, muscle biopsy in myositis

## Abstract

A 30-year-old woman presented with slowly progressive neck and proximal limb weakness over three years. Initial evaluation showed moderate creatine kinase elevation, anti-Mi-2β and anti-glycyl-tRNA synthetase (EJ) positivity, and electrophysiological and muscle biopsy findings interpreted as inflammatory myopathy. She was diagnosed with polymyositis and received multiple immunosuppressive therapies without clinical improvement. Despite normalization of creatine kinase, weakness and muscle wasting progressed. Re-evaluation revealed profound generalized atrophy, hyporeflexia, widespread limb and tongue fasciculations, and preserved sensation. Nerve conduction studies showed markedly reduced motor amplitudes with relative preservation of sensory responses, while repeat muscle biopsy demonstrated severe chronic neurogenic atrophy. Genetic testing identified a heterozygous FIG4 p.Arg197Cys variant of uncertain significance. Lower motor neuron dysfunction was demonstrated in multiple anatomical regions, and the overall clinical and electrophysiological findings were consistent with the Gold Coast diagnostic framework for amyotrophic lateral sclerosis with a lower-motor-neuron-predominant phenotype. Immunosuppression was withdrawn, weakness continued to progress, and mechanical ventilation was required approximately six months later. This case highlights the need to reconsider the diagnosis when presumed inflammatory myopathy progresses despite biochemical improvement and adequate immunosuppressive therapy, particularly when neurogenic features emerge.

## Introduction

Progressive muscle weakness is usually approached by first distinguishing a primary myopathy from a neurogenic disorder. Inflammatory myopathies typically cause predominantly proximal weakness, whereas lower motor neuron disorders more often feature muscle wasting, fasciculations, reduced reflexes, preserved sensation, and neurogenic motor-unit changes [1]. In practice, however, this distinction may be difficult, particularly early in the disease course, when clinical, biochemical, electrophysiological, and pathological findings may overlap. Mild creatine kinase (CK) elevation, spontaneous activity on electromyography, edema-like signal abnormalities on magnetic resonance imaging, and even limited inflammatory changes on muscle biopsy are not exclusive to inflammatory myopathies.

Myositis-specific antibodies (MSAs) have become important tools for the classification of idiopathic inflammatory myopathies (IIMs). Antibodies such as anti-Mi-2 and anti-aminoacyl-tRNA synthetase antibodies, including anti-EJ, are generally considered highly specific for IIMs and may strongly influence diagnostic and therapeutic decisions [2]. However, their diagnostic value is not absolute. MSAs have also been detected in patients with alternative neurological and neuromuscular disorders, particularly when commercial multiplex assays are used outside a strongly compatible clinical phenotype [3]. Therefore, antibody positivity should support, rather than independently establish, a diagnosis of inflammatory myopathy.

Motor neuron disease may be accompanied by muscle weakness and modest CK elevation, occasionally resulting in diagnostic confusion with inflammatory myopathy [4]. Diagnostic confusion may be further increased when early electromyographic or muscle biopsy findings are nonspecific. Failure to recognize an evolving neurogenic process may lead to prolonged and ineffective immunosuppressive treatment.

We report a young woman initially diagnosed with polymyositis based on positive MSAs and findings interpreted as inflammatory myopathy, whose subsequent clinical course and comprehensive neuromuscular reevaluation supported a diagnosis of lower-motor-neuron-predominant amyotrophic lateral sclerosis (ALS) under the Gold Coast framework [5].

## Case presentation

A previously healthy 30-year-old woman developed slowly progressive muscle weakness over three years. Her initial symptom was weakness of the neck muscles, characterized by rapid neck fatigability and difficulty maintaining head posture for prolonged periods. Over time, the weakness gradually progressed to involve both upper and lower limbs, with increasing involvement of the neck muscles. She reported no sensory symptoms or dysphagia. There was no relevant family history.

During the early course of the disease, she was evaluated at a major tertiary medical center. Initial laboratory testing revealed an elevated CK level of 600 U/L (normal range <200 U/L in females). A myositis antibody panel performed using a commercial multiplex line-blot immunoassay demonstrated anti-Mi-2β and anti-glycyl-tRNA synthetase (EJ) reactivity, both reported as 1+ on the semiquantitative scale; no confirmatory testing using an independent method was performed. An initial muscle biopsy showed variation in fiber size, scattered degenerating fibers, mild endomysial fibrosis, and sparse mononuclear inflammatory infiltrates. An electromyographic (EMG) study performed at the referring institution was reported as compatible with inflammatory myopathy; however, the original tracings were unavailable for subsequent independent review.

According to discharge summaries, investigation reports, and treatment records from the referring institutions, the original diagnosis of polymyositis was based on the combined findings of progressive predominantly muscle weakness, CK elevation, anti-Mi-2β and anti-EJ reactivity, the EMG interpretation, and the muscle biopsy abnormalities. She initially received pulse corticosteroid therapy followed by cyclosporine 200 mg/day and methotrexate 12.5 mg/week for approximately one year. Despite treatment, muscle weakness progressively worsened.

Approximately a year later, she was referred to another tertiary center and received a further course of pulse corticosteroid therapy followed by gradual tapering. Muscle strength improved only minimally and subsequently deteriorated again. She was later treated at a third referral center with two courses of rituximab, followed by maintenance low-dose corticosteroids and mycophenolate mofetil. No meaningful or sustained improvement was observed, and weakness of the neck and all four limbs continued to progress. After approximately two years of immunosuppressive treatment overall, she was referred to our center for comprehensive diagnostic reevaluation.

At presentation, she was markedly cachectic, with profound generalized muscle wasting and a body mass index of only 14 kg/m². Severe atrophy involved the neck, shoulder girdle, pelvic girdle, and limb muscles. She had no heliotrope rash, Gottron papules or Gottron sign, photosensitive or other inflammatory skin eruptions, mechanic’s hands, Raynaud phenomenon, inflammatory arthritis, oral ulcers, or other systemic autoimmune manifestations. She also denied myalgia.

Neurological examination revealed severe predominantly proximal weakness, with muscle strength graded 3/5 in the upper limbs and 2/5 in the lower limbs, while distal strength was relatively preserved. Deep tendon reflexes were diffusely diminished, whereas sensory examination remained normal. No upper motor neuron signs were identified. Of particular significance, widespread fasciculations were observed in the limb muscles, together with tongue atrophy and tongue fasciculations. The combination of profound muscle wasting, hyporeflexia, fasciculations, and preserved sensation raised increasing concern for a lower motor neuron process rather than an active inflammatory myopathy.

Her serum CK, initially 600 U/L at the referring institution, subsequently decreased to 230 U/L and eventually normalized to 40 U/L despite continued deterioration in muscle strength and worsening muscle atrophy. Muscle magnetic resonance imaging demonstrated diffuse muscle atrophy with patchy edema-like signal abnormalities involving the shoulder girdle, paraspinal muscles, and lower limbs, as illustrated in Figure 1.

**Figure 1:**
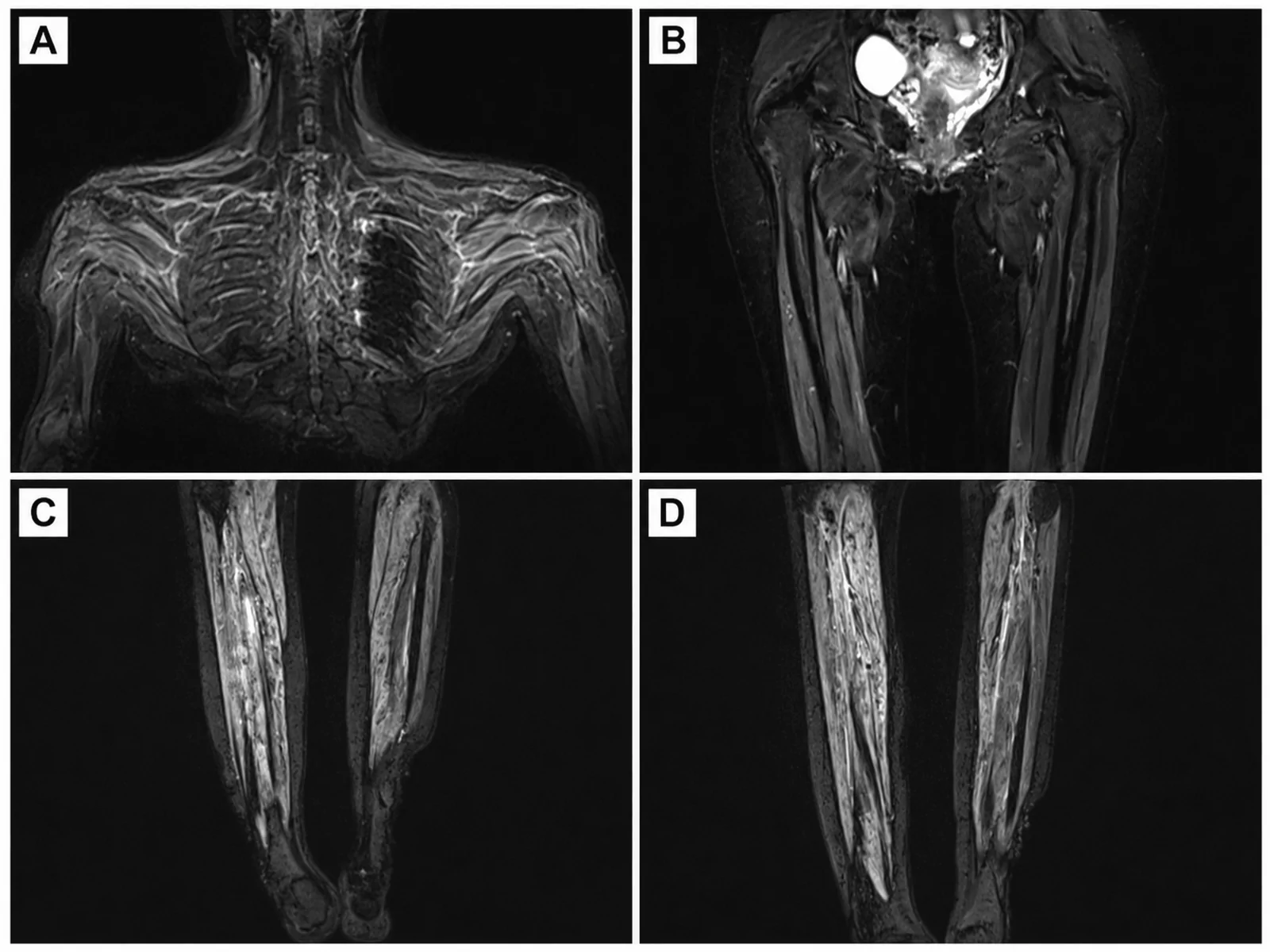
Coronal fluid-sensitive fat-suppressed (TIRM) muscle MRI (A) Bilateral patchy edema-like signal abnormalities in the shoulder girdle and paraspinal muscles with reduced muscle bulk; (B) Patchy bilateral edema-like signal abnormalities involving multiple thigh muscle groups with generalized atrophy; (C–D) Two different coronal images of the lower legs demonstrating extensive bilateral edema-like signal abnormalities and marked muscle atrophy, included to illustrate the extent and distribution of lower-leg involvement. Overall, MRI demonstrated widespread multifocal edema-like signal abnormalities on a background of severe generalized muscle wasting. These abnormalities are not specific for inflammatory myopathy and may also occur secondary to denervation.

A second open muscle biopsy of the rectus femoris revealed severe muscle fiber atrophy with grouped fiber loss and fatty replacement, findings consistent with chronic denervation, as shown in Figure 2.

**Figure 2:**
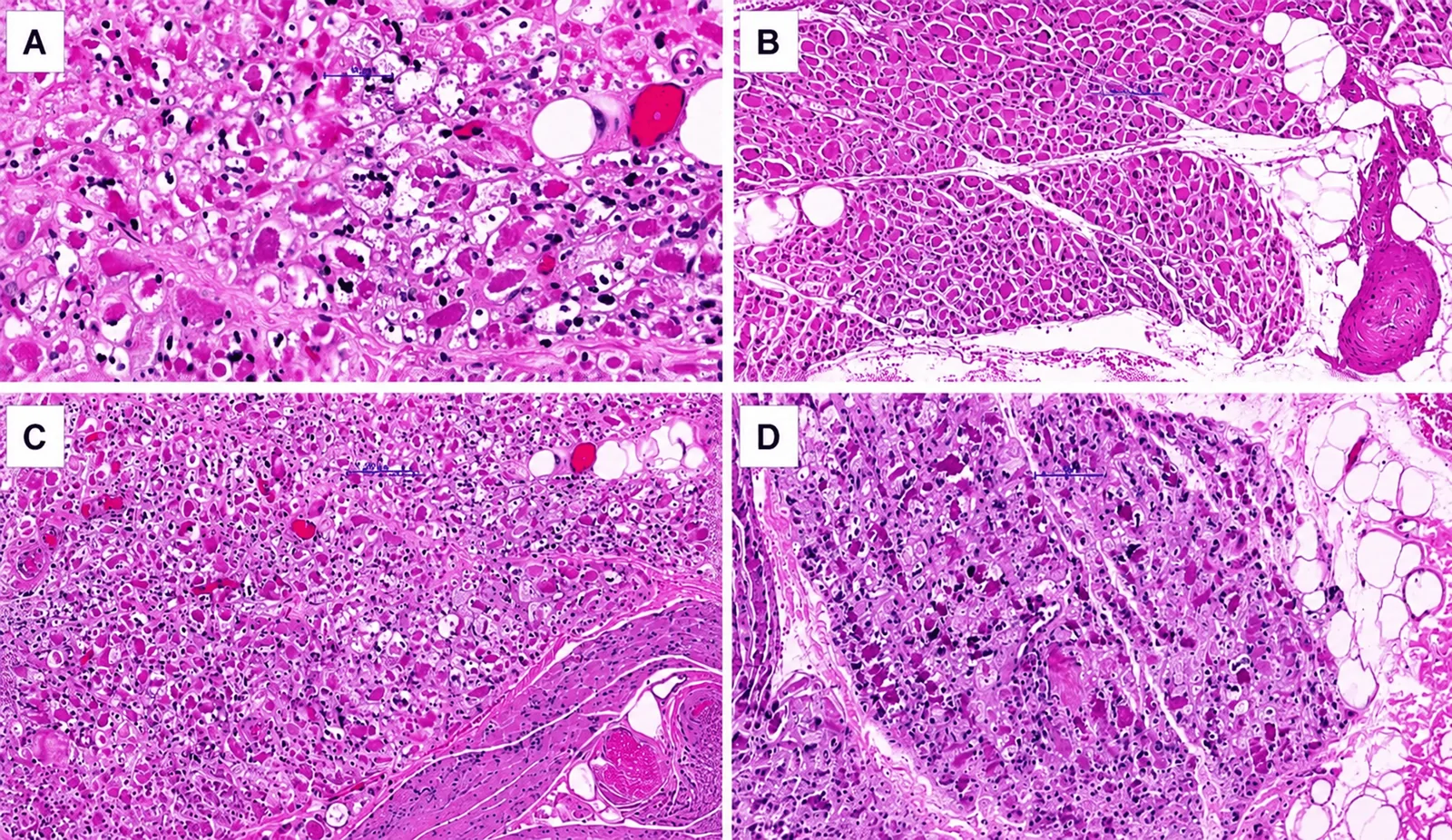
Histopathological findings of the repeat rectus femoris muscle biopsy (hematoxylin and eosin stain) (A) High-power view showing marked variation in muscle fiber size, with numerous small, angulated, severely atrophic fibers and prominent endomysial fibrosis (×400); (B) Low-power view of one fascicle demonstrating widespread grouped fiber atrophy with fiber loss and fatty replacement (×100); (C) Low-power view of a separate fascicle showing a similar pattern of extensive grouped fiber atrophy with endomysial fibrosis and fatty replacement, confirming that these changes are diffuse throughout the specimen (×100); (D) Intermediate-power view highlighting clusters of severely atrophic, angulated fibers with prominent endomysial fibrosis(×200). Overall, the findings are consistent with severe chronic neurogenic atrophy.

Table 1 summarizes the nerve conduction study findings, which demonstrated markedly reduced compound muscle action potential amplitudes across multiple upper- and lower-limb motor nerves with relative preservation of sensory responses, supporting a motor-predominant neurogenic process with sensory sparing.

**Table 1:** Summary of nerve conduction study findings Sensory nerve action potentials (SNAP) were preserved in the median, ulnar, sural, and superficial peroneal nerves bilaterally, whereas compound muscle action potential amplitudes were markedly reduced in multiple upper- and lower-limb motor nerves, consistent with a motor-predominant neurogenic process with sensory sparing. No definite conduction block was identified across the tested motor nerve segments. Sensory distal latency represents onset latency. Amplitudes are reported as SNAP amplitude (µV) for sensory studies and distal CMAP amplitude (mV) for motor studies. ADM, abductor digiti minimi; AH, abductor hallucis; APB, abductor pollicis brevis; CMAP, compound muscle action potential; EDB, extensor digitorum brevis; L, left; NCS, nerve conduction study; R, right.

Nerve	Side	Distal latency (ms)	Amplitude	Conduction velocity (m/s)	Interpretation
Sensory NCS					
Median, digit II	R / L	3.13 / 2.97	54.9 / 55.8 µV	45 / 47	Preserved sensory responses
Ulnar, digit V	R / L	2.76 / 2.66	55.6 / 57.5 µV	51 / 53	Preserved sensory responses
Sural	R / L	3.02 / 2.66	37.1 / 32.3 µV	43 / 49	Preserved sensory responses
Superficial peroneal	R / L	3.65 / 3.44	17.4 / 13.0 µV	40 / 41	Preserved sensory responses
Motor NCS					
Median–APB	R / L	7.55 / 6.04	0.1 / 0.4 mV	36 / 46	Markedly reduced CMAP amplitudes with prolonged distal latencies and reduced motor conduction velocity.
Ulnar–ADM	R / L	4.43 / 5.31	1.6 / 1.1 mV	46 / 58	Reduced CMAP amplitudes with prolonged distal latencies and mild motor conduction slowing.
Peroneal–EDB	R / L	6.56 / 8.75	0.8 / 0.3 mV	32 / 36	Markedly reduced CMAP amplitudes with prolonged distal latencies and reduced motor conduction velocity.
Tibial–AH	R / L	5.36 / 6.20	0.2 / 2.9 mV	36 / 39	Reduced CMAP amplitudes, severe on the right, with reduced motor conduction velocity.
F-wave	Reduced persistence in several tested motor nerves, with prolonged minimum F-wave latencies documented in multiple nerves.				

Table 2 summarizes the needle electromyography findings, which demonstrated widespread active and chronic neurogenic changes in bilateral proximal and distal upper- and lower-limb muscles, including positive sharp waves and fibrillation potentials at rest, large-amplitude, prolonged-duration, polyphasic motor-unit potentials, and reduced recruitment during voluntary contraction, without evidence of a primary myopathic motor-unit pattern.

**Table 2:** Summary of needle electromyography findings

Needle EMG component	Findings
Muscles examined	Bilateral tibialis anterior, gastrocnemius, vastus medialis, rectus femoris, biceps brachii, and deltoid
Spontaneous activity at rest	Positive sharp waves and fibrillation potentials at multiple sampled sites, with positive sharp waves predominating.
Motor-unit potentials during slight contraction	Predominantly medium to long duration motor-unit potentials; virtually no short duration units; many complex/polyphasic prolonged-duration units
Recruitment during maximal contraction	Reduced recruitment/interference pattern with increased firing of large-amplitude, prolonged-duration, polyphasic motor-unit potentials
Overall interpretation	Widespread active and chronic neurogenic changes without evidence of a primary myopathic motor-unit pattern

Representative needle-EMG tracings from the left rectus femoris are shown in Figure 3.

**Figure 3:**
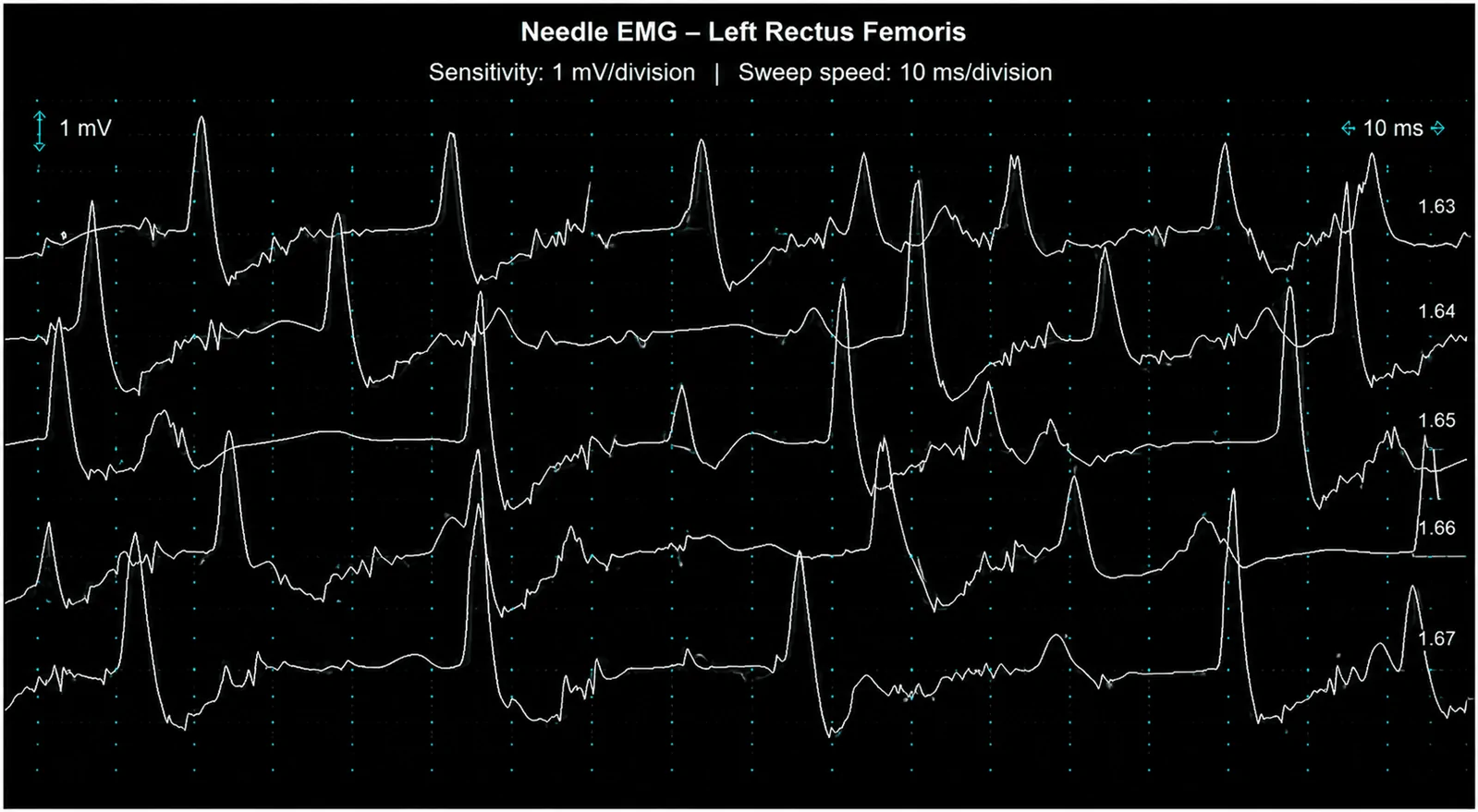
Representative needle electromyography recording from the left rectus femoris Representative needle electromyography tracing from the left rectus femoris during voluntary activation (sensitivity, 1 mV/division; sweep speed, 10 ms/division), demonstrating large-amplitude, complex motor-unit potentials with a neurogenic morphology.

Genetic testing using a motor neuron disease-related gene panel identified a heterozygous FIG4 c.589C>T (p.Arg197Cys) missense variant classified as a variant of uncertain significance. No functional or segregation evidence was available to establish pathogenicity. Accordingly, this finding did not establish a genetic diagnosis and was considered to be of uncertain clinical relevance.

No conduction block or abnormal temporal dispersion was identified on nerve conduction studies, and the available clinical and ancillary investigations revealed no alternative structural, inflammatory, or peripheral nerve disorder that better explained the progressive pure motor syndrome. Taken together, the progressive motor impairment, widespread fasciculations and muscle wasting, preserved sensation, and clinical and electrophysiological evidence of lower motor neuron dysfunction involving multiple anatomical regions fulfilled the Gold Coast diagnostic criteria for ALS, with a lower-motor-neuron-predominant phenotype [5].

Immunosuppressive therapy was subsequently discontinued, and management was redirected toward supportive neuromuscular care. Given the advanced progressive disease and the financial burden of prolonged tertiary-center treatment, the family elected a palliative-care approach and transferred the patient to a local hospital for ongoing care. Over the following six months, generalized weakness and muscle atrophy continued to progress, ultimately requiring mechanical ventilatory support. Follow-up information was subsequently obtained from the family. Detailed pulmonary function testing, respiratory muscle pressure measurements, arterial blood gas results, and other objective respiratory assessments from this period were unavailable because subsequent care occurred outside our institution.

## Discussion

This case illustrates an important diagnostic pitfall at the interface between inflammatory myopathy and motor neuron disease. Although the patient was initially treated as having polymyositis, longitudinal reassessment ultimately demonstrated lower-motor-neuron-predominant ALS. The progressive motor impairment together with clinical and electrophysiological evidence of lower motor neuron dysfunction involving multiple anatomical regions was consistent with the Gold Coast diagnostic framework for ALS [5]. The final diagnosis was therefore based on the evolving clinical and electrophysiological phenotype rather than on the genetic finding.

The original diagnosis of polymyositis reflected the convergence of findings that appeared supportive at the time but were individually nonspecific: proximal and neck weakness, moderate CK elevation, dual MSA reactivity, an outside EMG interpreted as myopathic, and sparse inflammatory changes on biopsy. In retrospect, the biopsy lacked the characteristic endomysial inflammation surrounding and invading non-necrotic fibers [6], while the original EMG tracings were unavailable for reassessment. Polymyositis should therefore be understood as the historical diagnosis of the referring institutions rather than one independently confirmed at our center.

The myositis antibody findings represented another potential source of diagnostic anchoring. Anti-Mi-2β and anti-EJ were detected using a commercial multiplex line-blot immunoassay and were both reported as 1+ on the semiquantitative scale; no confirmatory testing using an independent method was performed. Although MSAs are useful for the classification and phenotyping of idiopathic inflammatory myopathies, their diagnostic value depends on the clinical context and assay methodology [2]. In a recent cohort of 727 patients, Kleiser et al. found at least one myositis-specific or myositis-associated antibody in 14.6% of patients whose final diagnosis was not related to myositis [3]. Commercial line-blot assays may also yield clinically discordant results, particularly among weakly positive results and in patients with a low pre-test probability of inflammatory myopathy [7]. Discordance between commercial line-blot and immunoprecipitation methods has also been demonstrated for anti-Mi-2 and several antisynthetase antibodies [8]. In our patient, the absence of a characteristic anti-Mi-2 dermatomyositis phenotype or a clinically apparent antisynthetase syndrome further reduced the diagnostic weight of these findings. The dual 1+ antibody reactivity should therefore be regarded as supportive information rather than definitive serological evidence of inflammatory myopathy.

The biochemical and imaging findings were similarly nonspecific. Moderate CK elevation is well recognized in ALS and may occasionally overlap with levels observed in primary muscle disorders [4,9]. Electrophysiological studies have also suggested a relationship between CK elevation and denervation-related muscle injury in ALS [10,11]. In our patient, CK decreased from approximately 600 U/L to 230 U/L and eventually to 40 U/L despite continued progression of weakness and muscle wasting. This increasing dissociation between biochemical improvement and clinical deterioration was an important warning sign that ongoing inflammatory muscle injury was unlikely to explain the evolving phenotype.

Muscle MRI constituted another potential source of diagnostic confusion. Increased signal intensity on fluid-sensitive sequences is commonly interpreted as muscle edema and may support inflammatory myopathy; however, such abnormalities are not specific for inflammation. Denervated skeletal muscle may also develop edema-like signal abnormalities on fluid-sensitive MRI sequences [12]. Accordingly, the coexistence of patchy edema-like signal and marked generalized atrophy in this patient needed to be interpreted within the broader neurological phenotype rather than regarded in isolation as evidence of persistent myositis. In the setting of fasciculations, hyporeflexia, preserved sensation, and subsequent neurogenic electrophysiological findings, denervation provided a more coherent explanation for the MRI abnormalities.

Electrophysiological reassessment and repeat muscle pathology ultimately provided the strongest evidence for a neurogenic process. Fibrillation potentials and positive sharp waves may occur in both inflammatory myopathy and active denervation and therefore have limited diagnostic specificity when considered alone. Interpretation requires consideration of motor-unit morphology, recruitment, anatomical distribution, and evidence of chronic reinnervation. The Gold Coast criteria emphasize clinical or electrophysiological evidence of lower motor neuron dysfunction across appropriate anatomical regions in the setting of progressive motor impairment [5]. At reevaluation, our patient demonstrated widespread large-amplitude, prolonged-duration, polyphasic motor-unit potentials with reduced recruitment, together with markedly reduced motor responses and relative preservation of sensory responses. The repeat muscle biopsy showed grouped fiber atrophy, extensive fiber loss, fibrosis, and fatty replacement, findings consistent with chronic neurogenic injury [13]. Together, these findings provided a coherent explanation for the progressive pure motor phenotype and the lack of response to immunosuppressive therapy.

The Factor-Induced Gene 4 (FIG4) finding should be interpreted separately from the clinical diagnosis. Rare heterozygous FIG4 variants have been reported in ALS cohorts, including individuals with slowly progressive or lower-motor-neuron-predominant phenotypes [14,15]. However, the heterozygous FIG4 c.589C>T (p.Arg197Cys) variant identified in our patient remains classified as a variant of uncertain significance. No functional or segregation evidence was available to support pathogenicity, and interpretation of variants detected through ALS genetic panels requires caution [16]. Accordingly, this variant cannot be considered disease-causing, does not establish a diagnosis of FIG4-associated ALS, and was not used to establish the clinical diagnosis. The genetic etiology in this patient therefore remains unproven.

The principal limitations of this report are the inability to retrospectively review the original EMG tracings, reliance on a single semiquantitative antibody assay without independent confirmation, lack of objective respiratory physiological data during the later disease course, and the unresolved clinical significance of the FIG4 variant.

The central clinical lesson is the importance of longitudinal diagnostic reassessment when the disease course becomes inconsistent with the presumed diagnosis. Continued motor deterioration despite biochemical improvement and substantial immunosuppressive therapy should prompt renewed neurological and electrophysiological evaluation rather than automatic escalation of immunotherapy. In diagnostically discordant cases, repeat clinical, electrophysiological, and pathological assessment may be decisive in redirecting the diagnosis and avoiding prolonged ineffective treatment.

## Conclusions

Overall, this case demonstrates how moderate CK elevation, myositis antibody positivity, electrophysiological abnormalities, and limited inflammatory changes on muscle biopsy can collectively mimic inflammatory myopathy. The most important diagnostic clue was not a single investigation but the longitudinal discordance between the presumed diagnosis and the clinical course. Progressive weakness despite normalization of CK and intensive immunosuppressive treatment, particularly when accompanied by severe atrophy, hyporeflexia, fasciculations, and preserved sensation, should prompt reconsideration of a neurogenic disorder before inflammatory myopathy is labeled as treatment-refractory. MSA positivity and edema-like muscle MRI abnormalities should therefore be interpreted in the context of the overall clinical and electrophysiological phenotype rather than as disease-defining findings in isolation. Repeat neurological examination, electrophysiological assessment, and when appropriate, muscle pathology may help redirect the diagnosis and avoid prolonged ineffective immunosuppression. This case highlights the importance of longitudinal diagnostic reassessment when the evolving phenotype becomes inconsistent with the original diagnosis.
